# 1,25-Dihydroxyvitamin D_3_ inhibits the proliferation of rat mesangial cells induced by high glucose via DDIT4

**DOI:** 10.18632/oncotarget.23063

**Published:** 2017-12-09

**Authors:** Da-Peng Chen, Ye-Ping Ma, Li Zhuo, Zheng Zhang, Gu-Ming Zou, Yue Yang, Hong-Mei Gao, Wen-Ge Li

**Affiliations:** ^1^ Graduate School of Peking Union Medical College, Beijing 100730, China; ^2^ Department of Nephrology, China-Japan Friendship Hospital, Beijing 100029, China; ^*^ These authors share co-first authorship

**Keywords:** diabetic nephropathy, 1,25-Dihydroxyvitamin D_3_, proliferation, DDIT4, mTOR

## Abstract

1,25-Dihydroxyvitamin D_3_(1,25(OH)_2_ D_3_) is a secosteroid with antiproliferative property. It also plays a pivotal renoprotective role in diabetic nephropathy. We investigated whether 1,25(OH)_2_D_3_ could inhibit the proliferation of rat mesangial cells exposed to high glucose via the DNA-damage-inducible transcript 4/mammalian target of rapamycin(DDIT4/mTOR) pathway. The cell proliferation rate and cell cycle duration were measured using cell counting kit-8 assay and flow cytometry. Protein expression was assayed by Western blot. Glucose acted as a growth factor in rat mesangial cells, promoted cell proliferation. In parallel, the protein expression of DDIT4, TSC1/TSC2, and 4E-BP1 were decreased, and Rheb, mTOR, and p70S6K were increased. Acting via the DDIT4/mTOR signaling, 1,25(OH)_2_ D_3_ treatment reversed these pathological changes, upregulated DDIT4, TSC1/TSC2, and 4E-BP1, downregulated Rheb, mTOR, and p70S6K. The short-term overexpression of DDIT4 inhibited the proliferation of rat mesangial cells, similar to 1,25(OH)_2_ D_3_ treatment. siRNA knockdown of DDIT4 suppressed antiproliferative responses to 1,25(OH)_2_ D_3_. These results suggest that 1,25(OH)_2_ D_3_ inhibits the proliferation of rat mesangial cells induced by high glucose via the DDIT4/mTOR signaling pathway.

## INTRODUCTION

Diabetic nephropathy (DN) is one of the most common complications of type 1 and type 2 diabetes and the leading cause of end-stage renal disease in the Western world [1]. Proliferation of mesangial cells(MCs) and extracellular matrix(ECM) expansion have been considered as contributing factors to the initial pathophysiologic mechanisms involved in glomerulosclerosis, which is typical of DN [2, 3]. Thus, finding effective approaches to inhibit MCs proliferation is important for preventing glomerulosclerosis in patients with diabetic nephropathy.

1,25-Dihydroxyvitamin D_3_ (1,25(OH)_2_ D_3_), the hormonal form of vitamin D, is a member of the secosteroid hormone family whose actions extend far beyond its classic role in calcium homeostasis and bone metabolism. Many studies have demonstrated that 1,25(OH)_2_ D_3_ modulates cell growth and differentiation, including mesangial cells and podocytes [4–7]. The functions of 1,25(OH)_2_ D_3_ are mediated by the interaction of the vitamin D receptor (VDR) with the retinoid X receptor, which binds to specific vitamin D response elements in the promoter region of target genes, resulting in the inhibition of proliferation and the stimulation of differentiation [8].

The serine/threonine kinase mammalian target of rapamycin (mTOR) regulates cell growth, metabolism, and autophagy to maintain cellular homeostasis [9, 10]. The protein kinase mTOR exists in two distinct protein complexes: mTOR complex 1 (mTORC1) and mTORC2. mTORC1 regulates cell growth and proliferation by directly phosphorylating two regulators of translation, p70-S6 kinase (p70S6K) and 4E binding protein 1 (4E-BP1) [11]. The mTOR activation plays a pivotal role in the development of DN [12]. Hyperglycemia and its associated growth factors activate mTOR primarily through the phosphatidylinositol 3-kinase/Akt signaling pathway. The induction of mTORC1 by Akt leads to the phosphorylation and thus the inhibition of TSC1/TSC2, thereby stimulating the mTORC1 activator Rheb and leading to downstream effects on protein synthesis and cell proliferation [13, 14]. The role of mTORC2 in regulating cellular processes is not well understood.

The gene encoding DNA-damage-inducible transcript 4 (DDIT4, also known as REDD1) is highly conserved, from Drosophila to humans. The 25 kDa DDIT4 protein is transcriptionally upregulated in response to hypoxia and other cellular insults, including DNA damage, endoplasmic reticulum stress, and energy stress [15, 16]. Recent studies have shown that the binding of 1,25(OH)_2_D_3_ to the VDR can increase the expression of DDIT4, which can then activate TSC1/TSC2,thereby inhibiting the expression of mTOR [17–20]. As an essential regulator of mTOR activity, DDIT4 regulates cell growth, apoptosis, and autophagy but there have been few studies of its effects on MCs. Therefore, our study examined the effects of 1,25(OH)_2_ D_3_ on RMCs exposed to high glucose. It also sought to determine whether the effect was mediated by the DDIT4/mTOR signaling pathway.

## RESULTS

### 1,25(OH)_2_ D_3_ inhibits RMCs proliferation induced by high glucose

**Figure 1:**
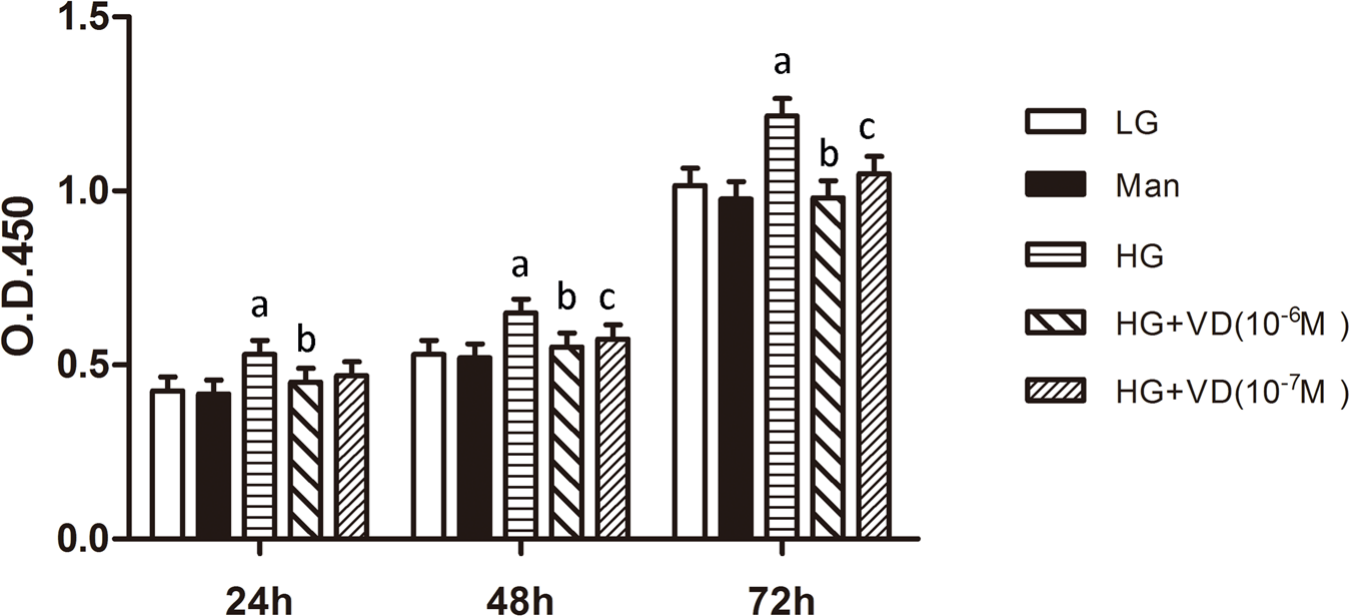
1,25(OH)_2_ D_3_ inhibits RMCs proliferation induced by high glucose The proliferative activity of RMCs was detected using a cell counting kit-8 assay. ^a^*p* < 0.05, HG vs. LG (*n* = 3); ^b^*p* < 0.05, HG + VD (10^−6^ M) vs. HG (*n* = 3); ^c^*p* < 0.05, HG + VD (10^−7^) vs. HG (*n* = 3). LG: 5.5 mM glucose; Man: 5.5 mM glucose + 24.5 mM mannitol; HG: 30 mM glucose.

The proliferative activity of RMCs cultured in the presence or absence of 1,25(OH)_2_ D_3_ was determined using the cell counting kit-8 assay. Cell proliferation was promoted by high glucose and significantly reduced by 1,25(OH)_2_ D_3_ (Figure 1). There were no significant differences between RMCs treated with 10^−6^ M and 10^−7^ M 1,25(OH)_2_ D_3_. The optimum response was obtained with 10^−7^ M, which was thus used in subsequent experiments.

### 1,25(OH)_2_ D_3_ regulates the cell-cycle distribution and cell size of RMCs treated with high glucose

**Figure 2:**
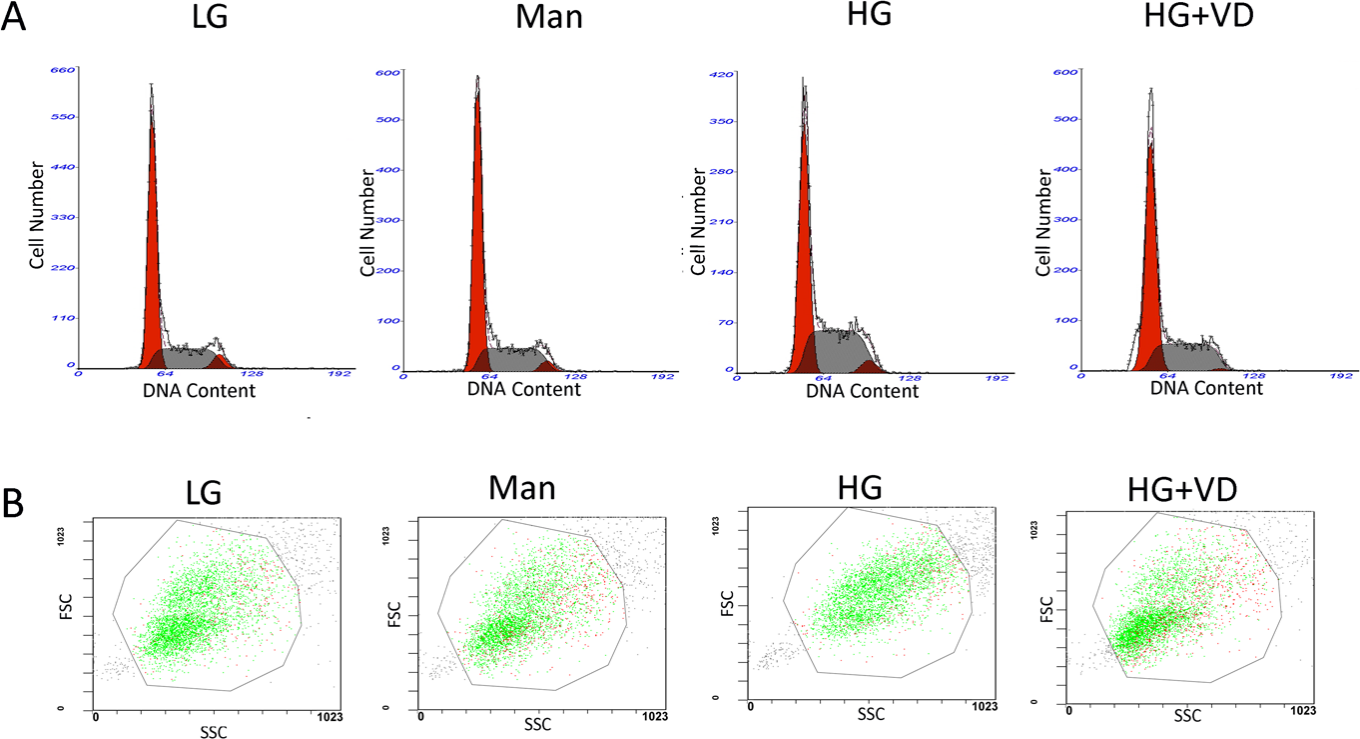
1,25(OH)_2_D_3_ regulates the cell-cycle distribution and cell size of RMCs treated with high glucose The cells were cultured for 48 h in the presence or absence of 1,25(OH)_2_ D_3_. (**A**) Cell cycle analysis. (**B**) Cell size. LG: 5.5 mM glucose; Man: 5.5 mM glucose + 24.5mM mannitol; HG: 30 mM glucose.

The effects of 1,25(OH)_2_D_3_ on the cell-cycle distribution and cell size of RMCs were examined using flow cytometry. High glucose induced a 18.5% decrease in the G0/G1 phase and a 41.9% increase in the S phase, indicating that high glucose promotes cell-cycle progression. Compared with the high-glucose group, 1,25(OH)_2_D_3_ markedly extended the G0/G1 phase and reduced the time spent by the cells in the S phase (Figure 2A and Table 1). 1,25(OH)_2_D_3_ also decreased the size of RMCs treated with high glucose (Figure 2B and Table 2).

**Table 1:** Cell-cycle distribution of RMCs

Group	G0/G1 (%)	S (%)	G2/M (%)
LG	63.6 ± 1.0	31.0 ± 0.8	5.4 ± 0.6
Man	63.2 ± 1.2	33.1 ± 1.0	3.7 ± 0.4
HG	51.8 ± 1.8^*^	44.1 ± 1.1^*^	4.1 ± 0.5
HG+VD	62.9 ± 1.7^#^	36.3 ± 1.0^#^	0.8 ± 0.06^#^

LG: 5.5 mM glucose; Man: 5.5 mM glucose + 24.5 mM mannitol; HG: 30 mM glucose.

^*^p < 0.05, HG vs. LG (*n* = 3), ^#^*p* < 0.05, HG+VD vs. HG (*n* = 3).

**Table 2:** Cell size of RMCs

Group	FSC
LG	511 ± 12
Man	509 ± 15
HG	581 ± 26^*^
HG+VD	480 ± 25^#^

LG: 5.5 mM glucose; Man: 5.5 mM glucose + 24.5 mM mannitol; HG: 30 mM glucose.

^*^p < 0.05, HG vs. LG (*n* = 3), ^#^*p* < 0.05, HG+VD vs. HG (*n* = 3).

### 1,25(OH)_2_ D_3_ modulates RMCs proliferation induced by high glucose via the DDIT4/mTOR signaling pathway

**Figure 3:**
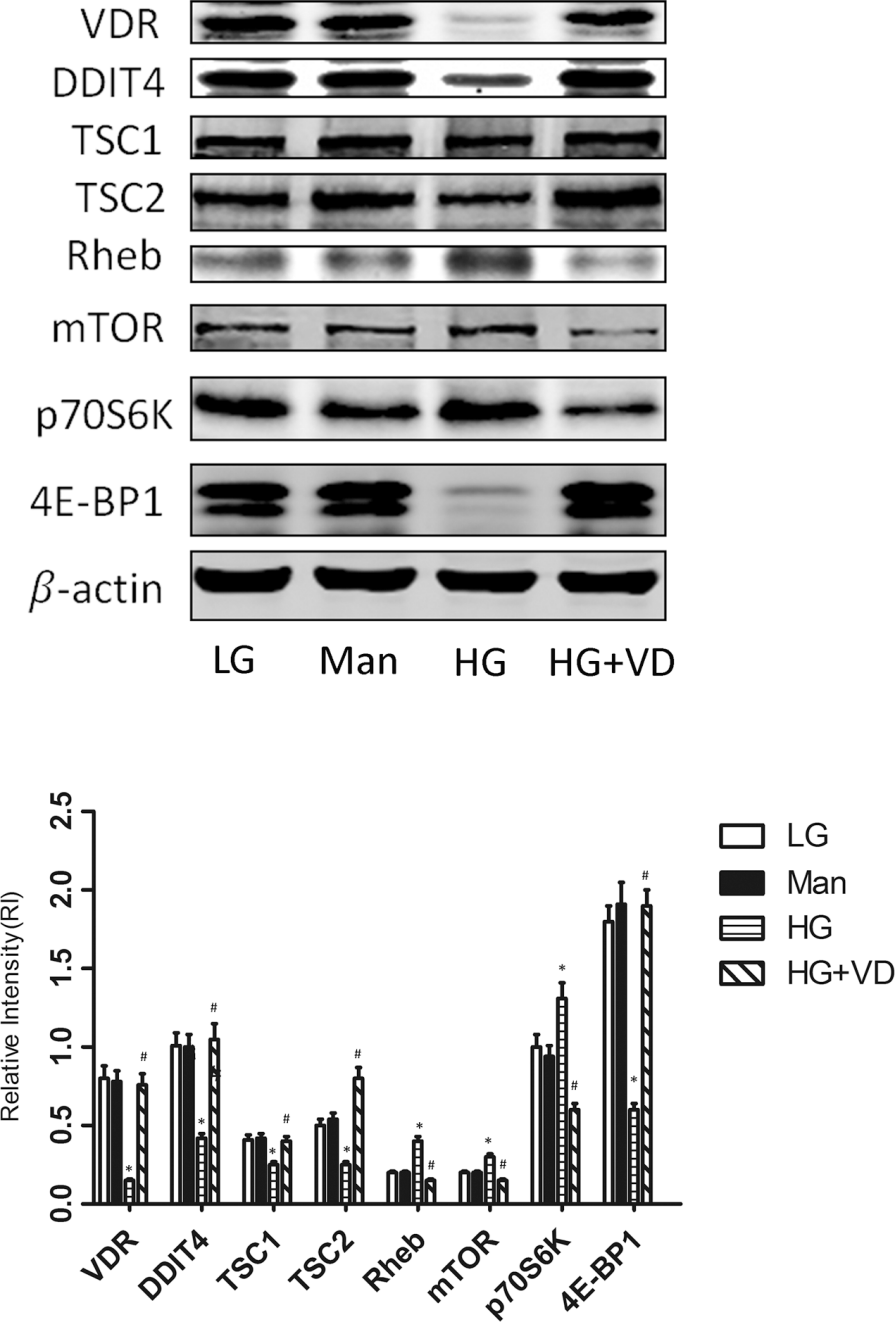
1,25(OH)_2_ D_3_ modulates RMCs proliferation induced by high glucose via the DDIT4/mTOR signaling pathway RMCs were cultured for 48 h in the presence or absence of 1,25(OH)_2_ D_3_. Cell lysates were subjected to Western blot analysis to measure the levels of the indicated proteins. The results are expressed as the relative intensity (adjusted to that of β-actin). ^*^p < 0.05, HG vs. LG (*n* = 3); ^#^ *p* < 0.05, HG+VD vs. HG (*n* = 3). LG: 5.5 mM glucose; Man: 5.5 mM glucose + 24.5 mM mannitol; HG: 30 mM glucose.

To investigate whether the DDIT4/mTOR signaling pathway is involved in regulating the proliferation of RMCs treated with 1,25(OH)_2_ D_3_ , we examined the expression of VDR, DDIT4, TSC1/TSC2, the mTOR mediator Rheb, mTOR, and its downstream proteins 4E-BP1 and p70S6K by Western blot. The protein levels of VDR, DDIT4, TSC1/TSC2, and 4E-BP1 were significantly downregulated (*p* < 0.05) and those of Rheb, mTOR, and p70S6K were significantly upregulated (*p* < 0.05) in the high glucose group vs. the low glucose group. Incubation of RMCs with 1,25(OH)_2_ D_3_ for 48 h increased VDR expression (*p* < 0.05), restored the expression of TSC1/TSC2 and 4E-BP1, and blocked the aberrant upregulation of Rheb, mTOR and p70S6K (Figure 3). These results suggest that the DDIT4/mTOR signaling pathway plays a key role in 1,25(OH)_2_ D_3_-modulated RMCs proliferation induced by high glucose.

### Short-term overexpression of DDIT4 suppresses RMCs proliferation via the mTOR signaling pathway

**Figure 4:**
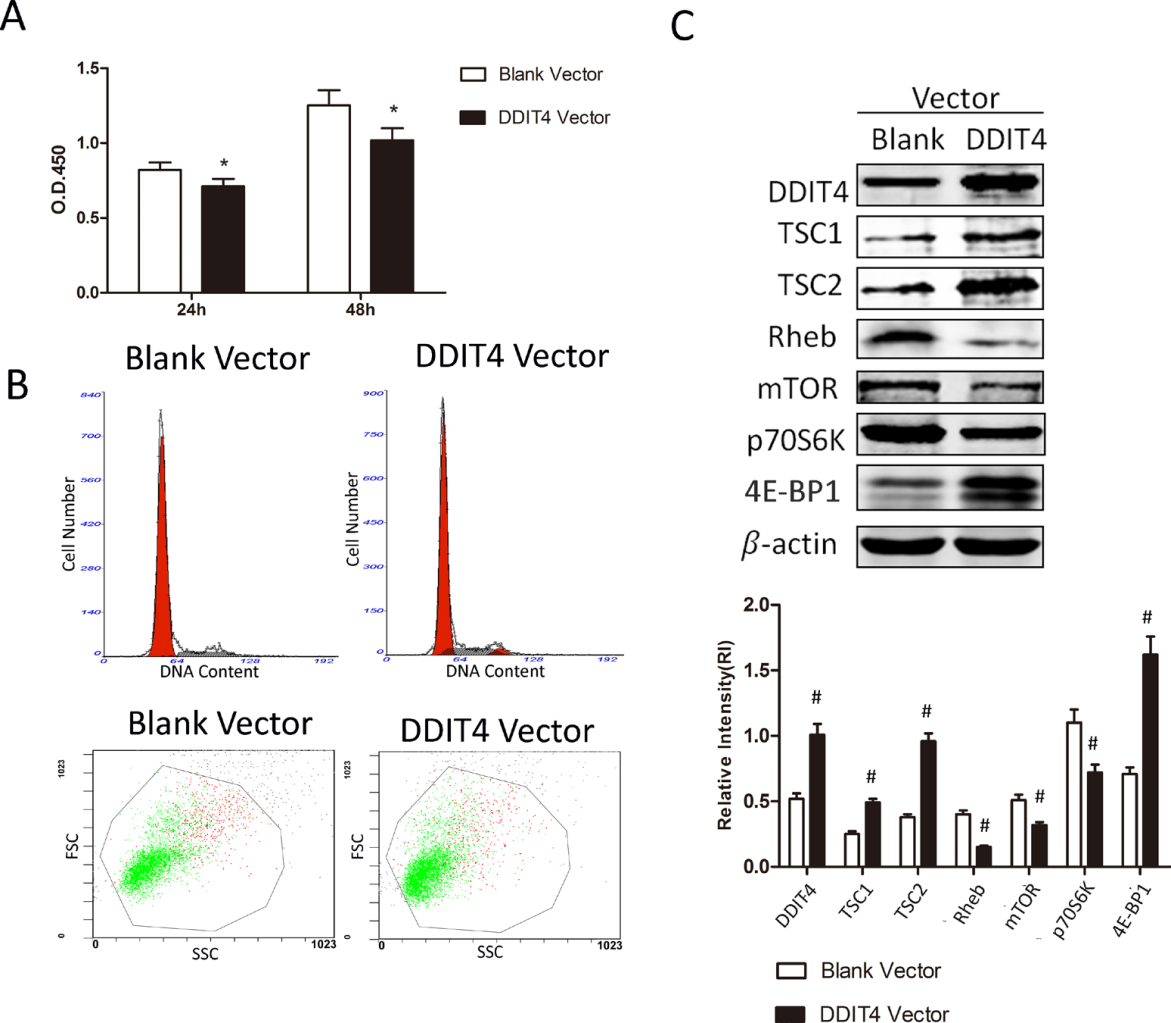
The short-term overexpression of DDIT4 suppresses RMCs proliferation and cell-cycle progression via the mTOR signaling pathway RMCs were transiently transfected with blank vector or DDIT4 lentiviral vector. (**A**) The proliferative activity of the transfected RMCs was detected using the cell counting kit-8 assay. ^*^p < 0.05, DDIT4 vector vs. blank vector (*n* = 3). (**B**) Analysis of the cell-cycle distribution and size of the transfected RMCs by flow cytometry. (**C**) DDIT4, TSC1/TSC2, Rheb, mTOR, 4E-BP1, and p70S6K protein expression levels were detected by Western blot. ^#^*p* < 0.05, DDIT4 vector vs. blank vector (*n* = 3).

RMCs were transiently transfected with blank vector or DDIT4 lentiviral vector. The short-term overexpression of DDIT4 suppressed RMCs proliferation and cell-cycle progression (Figure 4A and 4B, Tables 3 and 4). Western blotting showed the significant upregulation of TSC1/TSC2, 4E-BP1 (*p* < 0.05) and downregulation of Rheb, mTOR, and p70S6K (*p* < 0.05) in cells transfected with the DDIT4 vector (Figure 4C). Moreover, the results were similar to those obtained in 1,25(OH)_2_ D_3_-treated cells.

**Table 3:** Cell-cycle distribution of RMCs transfected with DDIT4 vector

Group	G0/G1 (%)	S (%)	G2/M (%)
Blank vector	87.1 ± 1.9	12.6 ± 1.3	0.3 ± 0.05
DDIT4 vector	81.8 ± 2.1^*^	14.5 ± 1.6	3.7 ± 1.0^*^

^*^p < 0.05, DDIT4 vector vs. blank vector (*n* = 3).

**Table 4:** Size of RMCs transfected with DDIT4 vector

Group	FSC
Blank vector	444 ± 12
DDIT4 vector	406 ± 14^*^

^*^p < 0.05, DDIT4 vector vs. blank vector (*n* = 3).

### siRNA knockdown of DDIT4 suppresses antiproliferative responses to 1,25(OH)_2_ D_3_

**Figure 5:**
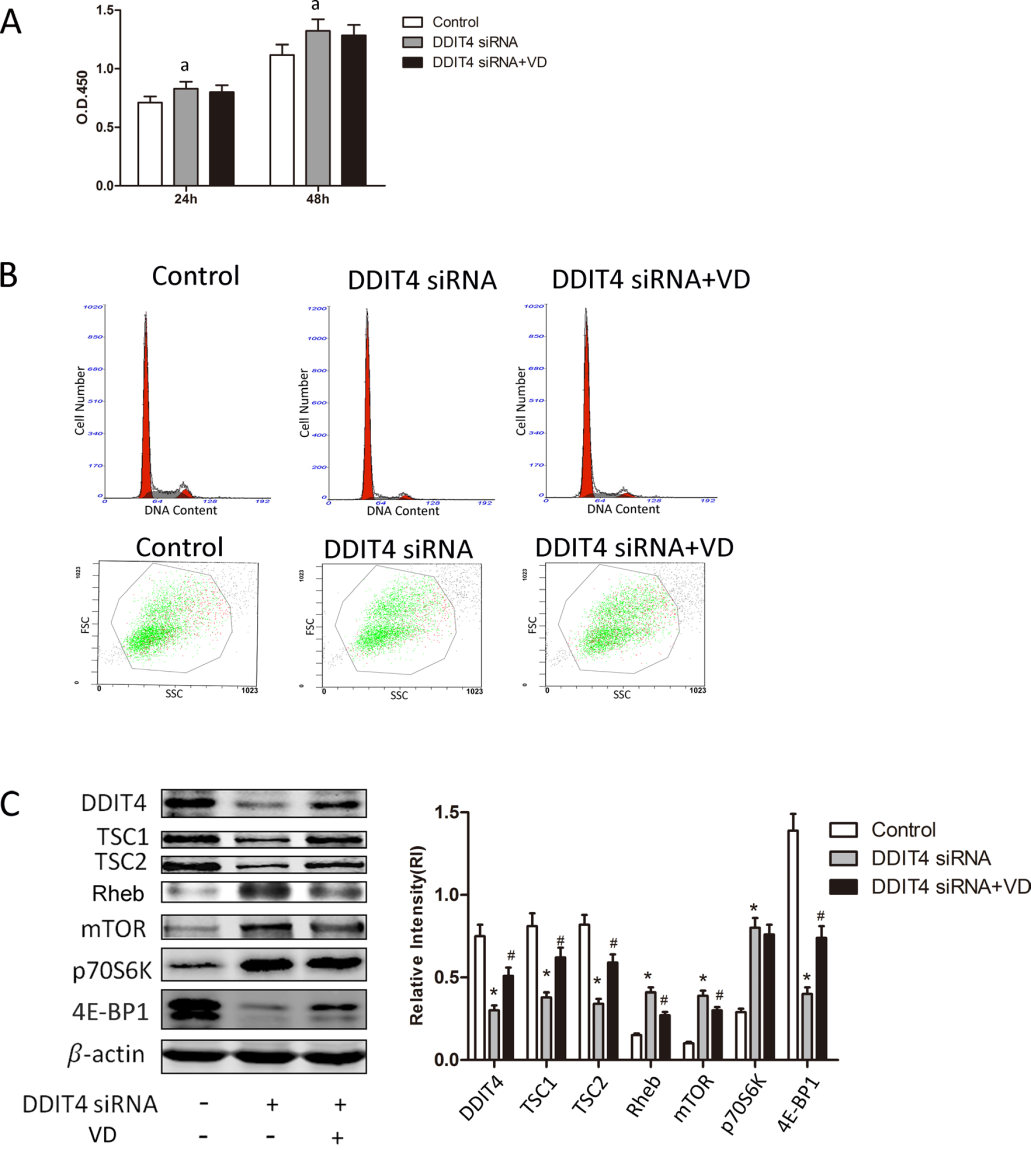
siRNA knockdown of the DDIT4 antiproliferative responses to 1,25(OH)_2_ D_3_ RMCs were transfected with negative control siRNA orDDIT4-specific siRNA in the presence or absence of 1,25(OH) _2_ D_3_ . (**A**) The proliferative activity of the transfected RMCs was detected using the cell counting kit-8 assay. ^a^*p* < 0.05, DDIT4 siRNA vs. control (*n* = 3). (**B**) Cell-cycle distribution and size of the transfected RMCs analyzed using flow cytometry. (**C**) DDIT4, TSC1/TSC2, Rheb, mTOR, 4E-BP1 and p70S6K protein expression levels were detected by Western blot. ^*^p < 0.05, DDIT4 siRNA vs. control; ^#^*p* < 0.05, DDIT4 siRNA+VD vs. DDIT4 siRNA (*n* = 3).

To determine the functional significance of DDIT4 in mediating RMCs responses to 1,25(OH)_2_ D_3_, siRNA was used to knock down the expression of DDIT4. DDIT4 mRNA expression was suppressed by 55% in cells exposed to DDIT4-specific siRNA but increased following cotreatment with 1,25(OH)_2_ D_3_, albeit not significantly (*p* > 0.05) . The level of DDIT4 protein was also suppressed in siRNA-treated RMCs (*p* < 0.05), whereas 1,25(OH)_2_D_3_ treatment specifically reversed DDIT4-siRNA-induced proliferation and hypertrophy (*p* > 0.05) (Figure 5A and 5B, Tables 5 and 6), restored DDIT4 and TSC1/TSC2 expression, and downregulated Rheb and mTOR expression (Figure 5C).

**Table 5:** Cell-cycle distribution of RMCs transfected with DDIT4 siRNA

Group	G0/G1	S	G2/M
Control	72.8 ± 1.8	20.2 ± 1.3	7.0 ± 1.0
DDIT4 siRNA	86.7 ± 2.1^*^	9.0 ± 1.0^*^	4.3 ± 0.06^*^
DDIT4 siRNA+VD	83.3 ± 2.1	12.4 ± 1.1^#^	4.3 ± 0.06

^*^p < 0.05, DDIT4 siRNA vs. control; ^#^*p* < 0.05, DDIT4 siRNA+VD vs. DDIT4 siRNA (*n* = 3).

**Table 6:** Size of RMCs transfected with DDIT4 siRNA

Group	FSC
Control	479 ± 17
DDIT4 siRNA	534 ± 24^*^
DDIT4 siRNA+VD	511 ± 21

^*^p < 0.05, DDIT4 siRNA vs. control.

## DISCUSSION

The pathogenesis of DM is complicated, the exact pathogenesis remain unclear. Advanced renal glycation end products [21, 22], the renin-angiotensin system (RAS) activation [23, 24], inflammation [25], and oxidative stress [26] have been shown to involve in DN. Previous studies have shown that vitamin D deficiency is a potential risk factor for diabetic nephropathy [27, 28]. In a randomized controlled trial, paricalcitol, an activated vitamin D analog, significantly reduced albuminuria in patients with diabetic nephropathy [29]. Glomerular basement membrane (GBM) thickening, ECM expansion, and MCs proliferation have long been recognized as pathological hallmark of diabetic nephropathy [3]. However, less is known about the effects of 1,25(OH)_2_D_3_ on MCs proliferation induced by high glucose and the molecular mechanisms involved in that process.

1,25-Dihydroxyvitamin D_3_ is an endocrine hormone with multiple physiological functions, including a pivotal role in immunomodulation and the inhibition of proliferation. [30]. 1,25(OH)_2_ D_3_ inhibited cell proliferation has been reported frequently in oncological studies, but this effect has seldom been shown in DN. Consistent with previous results [31], our *in vitro* study showed that high glucose promoted the proliferation of RMCs. We also found that cells treated with 1,25(OH)_2_ D_3_ had a significantly larger population in the G0/G1 phase and a smaller population in the S phase than cells cultured under high glucose. Thus, 1,25(OH)_2_ D_3_ could inhibit the proliferation of RMCs exposed to high glucose.

The mTOR activation plays a pivotal role in the development of DN [12, 32]. It has been demonstrated that mTOR regulates cell growth and proliferation by directly phosphorylating two direct downstream targets, p70S6K and 4E-BP1 [11]. Our study showed that the expression of mTOR and p70S6K was elevated in RMCs treated with high glucose. DDIT4 has been shown to inhibit cell growth via regulation of the mTOR signaling pathway upstream of the TSC1-TSC2 complex [17]. Lisse *et al.* [18] found that DDIT4 could act as a direct target of 1,25(OH)_2_D_3_ in the suppression of cell proliferation in response to vitamin D treatment in osteoblasts. Yang *et al.* reported that high glucose could inhibit the expression of DDIT4 whereas expression is restored by 1, 25(OH)_2_D_3_ treatment in β-cells [19]. Recently Wang *et al.* reported that *in vitro* and *in vivo* 1,25(OH)_2_D_3_ can effectively inhibit mesangial cells proliferation via the DDIT4/TSC2/mTOR pathway [20]. As predicted, the elevated expression of DDIT4 induced by 1,25(OH)_2_D_3_ was observed in the present study. Moreover, we found that the elevated expression of DDIT4 led to an increase in the expression of TSC1/TSC2, which result in the inhibition of mTOR expression. We provided evidence that 1,25(OH)_2_ D_3_ by directly promoting DDIT4 expression, regulated RMCs proliferation via the mTOR signaling pathway.

To obtain further evidence that 1,25(OH)_2_ D_3_ regulates the mTOR signaling pathway via DDIT4, RMCs were transfected with blank vector or DDIT4 lentiviral vector. The short-term overexpression of DDIT4 inhibited the proliferation RMCs . In the transfected cells, the level of DDIT4 protein was significantly upregulated whereas the levels of mTOR and the downstream protein p70S6K were downregulated, similar to the effects observed following 1,25(OH)_2_ D_3_ treatment.

siRNA (small interfering RNA) is able to regulate the expression of genes, by a phenomenon known as RNA interference [33]. siRNA has gained attention as a potential therapeutic reagent due to its ability to inhibit specific genes in many genetic diseases. It also can be used as tools to study single gene function both *in vivo* and *in vitro* [34]. RMCs were transfected with DDIT4-specific siRNA. siRNA knockdown of DDIT4 suppressed the antiproliferative responses of RMCs to 1,25(OH)_2_ D_3_ and extinguished the expression of mTOR, p70S6K, and 4E-BP1.

Taken together, our work provided strong evidence that *in vitro* 1,25(OH)_2_ D_3_ can inhibit the proliferation of RMCs induced by high glucose, by suppressing the mTOR signaling pathway, which is mediated by DDIT4 activation. In order to further clarify, our team will study further in animals.

## MATERIALS AND METHODS

### Reagents

Crystalline 1,25(OH)_2_ D_3_ (Sigma, St. Louis, MO, USA) was reconstituted in ethanol. 1,25(OH)_2_ D_3_ was added to the incubation medium to produce final medium concentrations ranging from 10^−7^ to 10^−6^ M.

### Cell culture and transfection

RMCs were obtained from the American Type Culture Collection (ATCC) and grown in RPMI-1640 medium containing 10% fetal bovine serum (FBS), 100 U penicillin/ml, and 100 µg streptomycin /ml in a 5% CO_2_ incubator at 37°C. Trypsin (0.25%) was used for cell passages. The cells were first synchronized in serum-free RPMI-1640 medium for 24 h, which was then replaced with DMEM containing 10% FBS and 5.5 mM glucose (low-glucose medium), DMEM containing 10% FBS, 5.5 mM glucose and 24.5 mM mannitol (mannitol medium), or DMEM containing 10% FBS and 30 mM glucose (high-glucose medium). Then all of the cells were cultured in the presence or absence of 1,25(OH)_2_D_3_. RMCs were transfected with the DDIT4 lentiviral vector (Applied Biological Materials Inc., Richmond, BC, Canada) or blank vector (Applied Biological Materials, Inc.) using Lipofectamine LTX and the Plus™ reagent (Invitrogen, Carlsbad, CA, USA). RMCs were transfected with DDIT4-specific siRNA (Applied Biological Materials, Inc.) or negative control siRNA (Applied Biological Materials, Inc.) using jetPRIME™ (Polyplus Transfection, lllkirch, France). The treated cells were assessed by cell counting kit-8 assay, flow cytometry, and Western blot.

### Cell proliferation assay

Cell proliferation was measured using the CCK-8 assay. RMCs were seeded in 96-well plates (4 × 10^3^ cells/well), synchronized by incubation in serum-free medium for 24 h, and then incubated with the test compounds as described above. After 24, 48, and 72 h, 10 µL CCK-8 reagent (Dojindo, Japan) was added to each well. The cells were cultured for 1 h, after which the optical density (OD) was measured at 450 nm using a microplate reader (Biotek, Winooski, VT, USA). The arithmetic mean OD of six wells per group was calculated.

### Flow cytometry

Cell-cycle analysis was performed using flow cytometry. RMCs were synchronized by incubation in serum-free medium for 24 h and then incubated with the test compounds for 48 h as described above. Then the cells were washed twice with cold phosphate-buffered saline (PBS) and fixed with 75% alcohol for 24 h at 4°C. The fixed cells were collected by centrifugation, washed with PBS, stained for 30 min at room temperature using the Coulter DNA prep reagent kit (Beckman Coulter, Inc., Brea, CA, USA), and finally analyzed using a Beckman Coulter FC 500 (Beckman Coulter, Inc.) together with the CXP software (Beckman Coulter, Inc.).

### Western blot

Proteins were extracted from RMCs using RIPA lysis buffer (50 mM Tris-HCl, pH 7.5, 150 mM NaCl, 0.5% deoxycholate, 1% Nonidet P-40, 0.1% SDS, 1 mM PMSF, and protease cocktail at 1 μg/mL). Protein concentrations were determined using a BCA kit (Thermo Scientific, Rockford, AL, USA). Equal amounts of protein (60 µg) were separated by SDS-PAGE on a 6%, 10%, or 12% acrylamide gel and then transferred onto a nitrocellulose membrane. After the membrane had been blocked with non-fat dry milk in Tris-buffered saline containing 0.05% Tween 20 for 1 h at room temperature, it was probed with the following primary antibodies overnight at 4°C: anti-VDR (catalogue no.: ab109234, Abcam, USA), anti-DDIT4 (catalogue no.: NBP1–77321, Novus Biologicals, USA), anti-TSC1 (catalogue no.: 6935S, Cell Signaling Technology, USA), anti-TSC2 (catalogue no.: 4308P, Cell Signaling Technology), anti-Rheb (catalogue no.: 13879S, Cell Signaling Technology), anti-mTOR (catalogue no.: Ab51044, Abcam), anti-4E-BP1 (catalogue no.: ab2606, Abcam), and anti-p70S6K (catalogue no.: ab32359, Abcam). After extensive washing, the membranes were incubated with Dylight anti-rabbit IgG secondary antibody. The antigens were visualized using the Odyssey infrared imaging system (LI-COR Biotechnology, Nebraska, USA). The results were expressed as the relative intensity (RI) intensity (adjusted to that of β-actin) of each band.

### Quantitative reverse-transcriptase polymerase chain reaction (qRT-PCR)

Total RNAs were isolated using TRIzol reagent (Invitrogen, USA). Total RNA (2 µg) was reverse-transcribed to obtain the cDNA using a TransScript first-strand cDNA synthesis SuperMix kit (TransGen Biotech, Beijing, China) according to the manufacturer’s instructions. Real-time PCR was performed in an Applied Biosystems 7500 real-time PCR system using a SYBR Select master mix kit (Applied Biosystems, Foster City, CA, USA). The PCR primers are shown in Table 7. The PCR conditions for all genes were as follows: initial denaturation at 95°C for 10 min followed by 40 cycles of denaturation at 95°C for 30 s; annealing at 59°C for 30 s; and extension at 72°C for 30 s. The relative RNA levels were calculated using the ΔΔCt method [35].

**Table 7:** Primers used in qRT-PCR

Gene	Direction	Primer
DDIT4	Forward	5′-TCTTGTCCGCAATCTTCGCT-3′
DDIT4	Reverse	5′-GGAGGACGAGAAACGATCCC-3′
18s	Forward	5′-GTAACCCGTTGAACCCCATT-3′
18s	Reverse	5′ -CCATCCAACGGTAGTAGCG-3′

### Statistical analyses

The results are expressed as the mean ± SD. Statistical analyses were performed using the SPSS 20.0 software package (SPSS, Inc., USA). Statistical comparisons between multiple groups were performed using a one-way ANOVA, applying the Bonferroni method to control for multiple testing. A *p* value < 0.05 was considered to indicate statistical significance.
